# Toronto ACHD program: A 65 year legacy

**DOI:** 10.1016/j.ijcchd.2024.100563

**Published:** 2024-12-30

**Authors:** J.M. Colman, W.G. Williams, C.K. Silversides, L. Harris, L. Benson, J. Heggie, R. Alonso-Gonzalez, E. Oechslin

**Affiliations:** a Toronto Adult Congenital Heart Disease (ACHD) Program, Peter Munk Cardiac Centre, University Health Network (UHN), Toronto, Canada; b Labatt Family Heart Centre, Hospital for Sick Children (SickKids), Toronto, Canada; c University of Toronto, Canada

## Abstract

The Toronto Adult Congenital Heart Disease (ACHD) Program at the University Health Network, University of Toronto, began in 1959. It traces its origins to a Paul Wood protégé, Dr. John Evans, and to a long-standing and supportive relationship with Hospital for Sick Children (SickKids), located just across the street. Over the decades, the program has grown to become a major center for training and research in ACHD and one of the largest clinical programs for ACHD care globally. This paper recounts the 65-year history of the program, including some of its key individuals, challenges, milestones, innovations, discoveries, and future aspirations.

## Teaser

The Toronto Adult Congenital Heart Disease (ACHD) Program at the University of Toronto, began in 1959. It traces its origins to a Paul Wood protégé and to a long-standing and supportive relationship with Hospital for Sick Children (SickKids). The program has grown to become a major center for training and research in ACHD and one of the largest clinical programs for ACHD care globally. This paper recounts the 65-year history of the program, including some of its key individuals, challenges, milestones, innovations, discoveries, and future aspirations.

## 1 The ACHD journey began at SickKids

The first SickKids cardiac surgeon, Dr. William Mustard, who had originally trained in general and orthopedic surgery, spent six weeks in Baltimore in 1947 observing Dr. Alfred Blalock and Vivien Thomas, L.L.D. (hon.) [1]. Upon returning to Toronto in 1948, Dr. Mustard became the first surgeon in Canada to perform extracardiac repairs such as ligation of patent ductus arteriosus, repair of coarctation of the aorta and Blalock-Taussig-Thomas shunt [2]. By 1950, the experimental monkey lung oxygenator offered the possibility of intra cardiac repair [3]. A series published in 1957 detailed 21 children in whom repair of congenital heart defects was attempted using the monkey lung oxygenator [4]. There were three survivors. Eventually this approach was abandoned, but it set the stage for future advances, and in 1957, soon after Dr. John Kirklin at the Mayo Clinic and Dr. C. Walton Lillehei at the University of Minnesota initiated the use of cardiopulmonary bypass for open heart surgery, this technique was applied in Toronto for an ASD repair. This led to progressive success with other simple lesions, then complex lesions such as Mustard operation for transposition of the great arteries in May 1963 [5], and then extremely complex lesions such as staged repair of hypoplastic left heart syndrome in 1982. By the year 2000, over 90 % of children born with congenital heart disease survived to adulthood. That same year, the number of adults living with CHD in Canada surpassed the number of children living with CHD, a difference that has progressively increased year over year since [2,6].

Other pioneering SickKids congenital cardiac surgeons were also critical to the development of the ACHD Program. After surgical training in Toronto, Dr. George Trusler, on a 6-month tour of North American medical centres, spent 2 weeks with Dr. C.W. Lillehei in Minneapolis in 1956. He wrote back to Dr. Mustard about that experience, motivating SickKids to buy the DeWall-Lillehei heart-lung bypass machine [7]. In 1976, Dr. Trusler partnered with Dr. William G. (Bill) Williams, who had been a cardiac surgery resident and fellow with Dr. Mustard toward the end of the latter's career. Dr. Williams went on to become head of cardiac surgery at SickKids and the first congenital heart surgeon appointed at Toronto General Hospital (TGH, now UHN), where he led the adult congenital cardiac surgical program until his retirement in 2006.

Even earlier, the paediatric cardiology program at SickKids began. Dr. John Keith was the first dedicated professor of Paediatric Cardiology anywhere, appointed at SickKids in 1938. In 1946, he was the first in Canada to use cardiac catheterization and angiography in children. He authored the first major textbook of pediatric cardiology [8]. Dr. Vera Rose became his fellow in 1955 and eventually a colleague. Dr. Rose was responsible for the development of a paper database (“Zebra sheets”) in the pre-computer era. The Zebra sheets, data from which had to be coded onto punch cards in a very labour-intensive manner, were the foundation for standardized data collection in CHD at SickKids and later at TGH and yielded much of the data for Keith's textbook. After mandatory retirement from an illustrious career as Professor of Pediatrics at SickKids, Dr. Rose joined the team at TGH, where she continued to train a new generation of fellows in ACHD for another 20 years. Dr. Keith was succeeded at SickKids by Dr. Richard (Dick) Rowe and then Dr. Robert (Bob) Freedom, the latter two integral in the early development of the Toronto ACHD program. Dr. Jeff Smallhorn in echocardiography and Dr. Marlene Rabinovitch in pulmonary hypertension also played significant roles.

Dr. Lee Benson joined the pediatric cardiology staff at SickKids in 1983 as director of the cardiac catheterization laboratory and became the first congenital interventional cardiologist. In addition to being a leader in pediatric congenital heart interventions, Dr Benson began a critical and continuing collaboration with the ACHD program at TGH in 1986.

## 2 The Toronto ACHD program – the early years

**Fig. 1:**
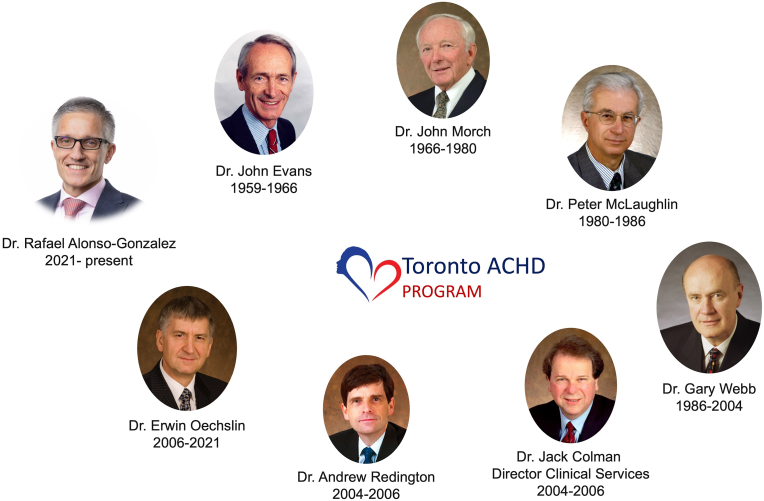
Directors of the Toronto ACHD Program, 1959 to current.

Dr. John Evans founded the Toronto ACHD clinic in 1959. He was joined by Dr. John Morch in 1966. Dr. Peter McLaughlin, an interventional cardiologist, was Dr. Morch's fellow and successor from 1980, passing the directorship to Dr. Gary Webb in 1986 (Fig. 1).

In the mid-1980's, transfers from SickKids began to increase, not just survivors with palliated cyanotic CHD and Eisenmenger syndrome as in the past, but now young adults who had been repaired for more complex congenital heart disease. By then, while maintaining his “day job” at SickKids as Chief of Cardiac Surgery Dr. Williams teamed with Dr. McLaughlin and Dr. Webb to establish the modern ACHD program. Dr. Williams recognized early that successes in pediatric congenital cardiac surgery and cardiology had created an expanding cadre of survivors who had had repairs or palliations but were not “fixed” and would need life-long medical and surgical care. He accepted the responsibility to ensure that the investment in CHD patients made during infancy and childhood was not abandoned during adulthood. Thus, he joined as a full partner with the ACHD medical team. Dr. Benson, congenital interventional cardiologist, shortly followed, also while continuing his role at SickKids, and partnered with Dr. McLaughlin in 1986 to found the ACHD cardiac interventional program. Dr. Eric Horlick joined Dr. McLaughlin and ultimately assumed responsibility for the interventional program with Dr. Benson’s ongoing collaboration and, later, that of Dr. Mark Osten.

**Fig. 2:**
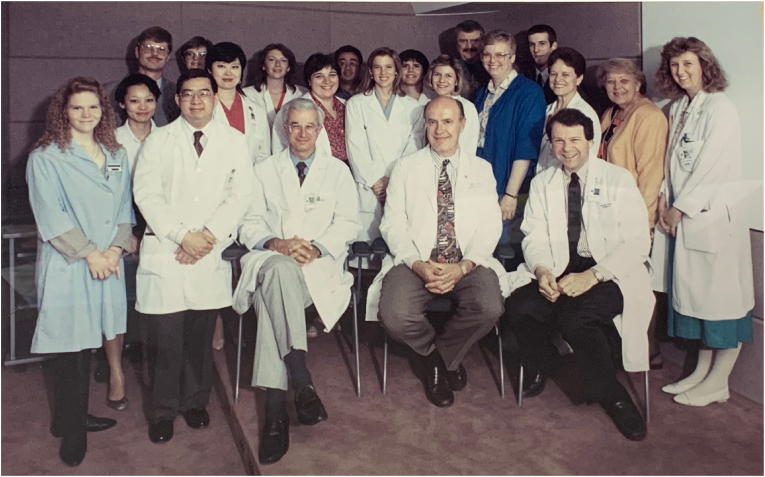
The first Toronto ACHD team picture, 1993. *First row:* Darlene Andrews^V^, Samuel Siu^C^, Peter McLaughlin^C^, Gary Webb^C^, Jack Colman^C^*Second row:* Theresa Sue^CO^, Lin De Yang^R^, Venera Bruto^P^, Susan Farrow^P^, Jane Irvine^P^, Eleanor Pakozdi^SE^, Adele Favero^N^, Audrey Liota^V^, Loretta Daniel^C^*Third row:* Stephen Dunn^C^, Bette Ross^R^, Janice Walters^R^, Jesse Cardona^V^, Suzanne Legault^P^, Leon Zelovitsky^RA^, Andrew Hurst^R^*Key:* C = Cardiologist; CO = Coordinator; N = Nurse; P = Psychosocial; R = Research; RA = Radiology; SE = Secretary; V = Volunteer.

The early pioneers from adult cardiology also accepted the challenge to be true to the children and families who were new survivors of what heretofore had been lethal conditions. As adults, CHD patients needed care, but what sort of care, and how much, and for how long, was a black box in the early days. Drs. Webb and McLaughlin were there from the beginning of the modern era. They were joined for some years by Dr. Peter Liu, who initiated an ACHD Symposium in Toronto in 1991. Dr. Jack Colman joined in 1988 and collaborated with Dr. Samuel Siu in 1992 to develop the Pregnancy and Heart Disease program (Fig. 2). More and more young women with CHD were reaching reproductive age at a time when their management and prognosis in pregnancy were a mystery. Dr. Louise Harris, who joined the ACHD team in about 1989, was one of the first electrophysiologists to focus on the care of the ACHD patient with arrhythmias. By 1989, Dr. John Evans’ CHD clinic from 1959 was restructured and reorganized into the ***Toronto Congenital Cardiac Centre for Adults (TCCCA)***, the name recently simplified to the current ***Toronto ACHD Program***. Starting as a small niche area of cardiology, with a few thousand registered patients and just over 500 clinic visits in 1989, then increasing in numbers, complexity and surgical profile by 1997 [9], the program has grown to care for more than 25,000 registered patients, seeing more than 6000 ACHD outpatients annually.

Aside from its early start, why was a program at Toronto General Hospital, in Canada, destined to become a world leader in ACHD organization, clinical care, research and training? It started with location. Although TGH and SickKids are independent institutions, with separate boards and foundations, they sit across a minor Toronto arterial road from each other. Both are teaching institutions of the University of Toronto. They operate in a Canadian medical context, where universal health care legislated by the Canada Health Act has been law of the land since 1984. SickKids has a firm policy that care is delivered only to patients under the age of 18 (as do all children's hospitals in Canada), obligating transfer to non-pediatric care thereafter. There was little or no incentive for non-ACHD cardiologists caring for adults to hold onto complex patients with conditions with which they were not familiar. Furthermore, the “eighteen and out” rule at pediatric hospitals precluded hospital-based pediatric cardiologists from continuing care. But in the early years there was little awareness of the existence of cardiologists outside pediatric centres with a mission to care for CHD patients as adults. An early (and ongoing) task of the ACHD program was to educate referring pediatric cardiologists and non-ACHD cardiologists about the program, and about the emerging understanding of the special needs of ACHD patients that could not be well-met in the community. Transitioning patients themselves were unaware of ACHD as a specialty of cardiology or, in many cases, of the importance of specialized life-long care. The Toronto ACHD program recognized this issue early [10]. Loss to follow-up of transferring patients was common until interdisciplinary and collaborative transition processes were established [[11], [12], [13]]. At SickKids, the importance of ACHD was recognized within the leadership, and strong collaboration to assist decision-making helped the ACHD program when expertise among ACHD practitioners was embryonic. Dr. Robert Freedom, as head of paediatric cardiology, and his successor Dr. Andrew Redington, provided unwavering support. In the 2004–2006 interregnum between Dr. Gary Webb's departure and the recruitment of Dr. Erwin Oechslin, Dr. Redington tag-teamed with Dr. Jack Colman to direct the ACHD program, ensuring its survival.

There is no doubt that the various components of the program in its early years were created, held together, expanded, and innovated, by one extraordinary personality. That was Dr. Gary Webb, director from 1986 to 2004. He set the patterns and the legacy of the Toronto program that continue today and brought the lessons learned in Toronto and the questions unanswered to a global audience. He led the development of the first Canadian Consensus Conference on Adult Congenital Heart Disease, presented in 1996 [14]. That document and its subsequent revisions served as a primary clinical guideline for ACHD management for many years. In 1991, he was one of the founders of the Canadian Adult Congenital Heart network, known as CACHnet (www.cachnet.org), the Canadian collaborative of ACHD and allied health practitioners. Then in 1994 he became the first president of the International Society for Adult Congenital Heart Disease (ISACHD, www.isachd.org). The mandate of both the Canadian and International organizations was to develop and sustain clinical and research networks in ACHD. In 1990, Dr. Joseph Perloff of UCLA had invited Dr. Webb to attend the 22nd American College of Cardiology (ACC) Bethesda Conference called “Congenital Heart Disease after Childhood” [15], an experience that catalyzed his future leadership in the field. In 2001 he co-chaired the next similar conference, the 32nd ACC Bethesda conference [16], which yielded a foundational document that identified the issues and directed the development of the field in the years thereafter. He was co-editor of the early comprehensive textbook, *Diagnosis and Management of Adult Congenital Heart Disease* [17], currently in its 4th edition, to which Toronto ACHD faculty contributed many chapters. His vision and passion to improve ACHD care, education and research enriched the ACHD world, and in so doing immeasurably enriched the program in Toronto where he began and eventually ended his career [18].

## 3 Some core elements of the Toronto ACHD program

With a foundation from 1959, a nurtured relationship with SickKids, a firm program structure established through the leadership of Dr. Gary Webb, dedicated commitment by the congenital heart surgeons led by Dr. Bill 10.13039/100001916Williams, and divisional and institutional leadership open to learning about and ultimately supporting this nascent field, the Toronto ACHD program continued to flourish. The origins of the Toronto program, in common with the London UK program at National Heart (now Royal Brompton) [19], the New Zealand program at Green Lane [20], the UCLA program led by Dr. Joseph Perloff, and others, can all trace a direct path back to Dr. Paul Wood at the National Heart Hospital in the mid 1950s. No doubt Dr. Wood would be pleased at the growth and success of the field he fathered.

### 3.1 Fellowship training

**Fig. 3:**
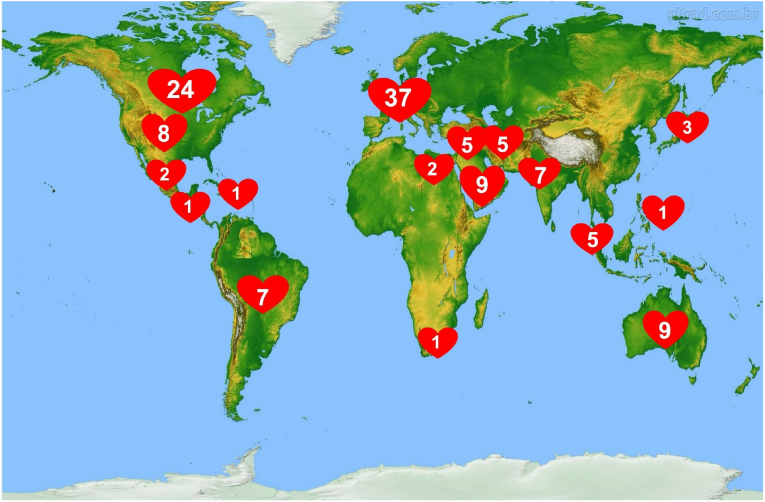
Region of origin of Fellows of the Toronto ACHD program since 1993. (See Appendix A for details.)

One of the most important goals of the Toronto ACHD program has been its longstanding commitment to training the next generation of ACHD cardiologists. From 1993 to 2024, the Toronto program has ***trained almost 130 fellows in ACHD*** who have enriched our own program, taken staff positions across Canada, and spread across the world (Fig. 3). (see also: Appendix A)

### 3.2 Collaboration

In the early years of care of adults with CHD, managing a population that had never previously existed, important benefits accrued from collaborative care. In contrast to experts in many other fields in which the body of knowledge and experience was better developed as a result of randomized trials and large observational studies, those who worked in ACHD depended greatly on consensus, on lessons from pediatric cardiology and congenital heart surgery, and on sharing responsibility for decisions when data were few and experience was limited. The benefits of multi-disciplinary conferences and of group review of inpatients on a regular basis was recognized early. To quote Dr. Bill Williams: “Management of congenital heart disease patients at any age is a team sport”. The culture supported extensive information- and responsibility-sharing, despite the cost in extra time in meetings. Working in such a milieu, it was easy to learn, and progressively to improve one's ability better to support, as adults, the babies and the children set on life's path by innovative surgery and interventions during infancy and childhood.

### 3.3 Database and standardized reporting

An early priority was to create a database on which research could be based and the patient population organized. Registry databases have subsequently become ubiquitous, but this was not so in the days when the SickKids Zebra sheets were designed. The Toronto program supported the design and implementation for the ACHD population of an early computer-based database (ultimately named CAPS), offering diagnostic granularity well beyond what could be found in, for example, ICD-9 codes, and the ability to capture as well testing details, demographics, appointments, and so on. The database was unique for its time in fulfilling the needs of ACHD clinics, such that its use spread widely in North America, Europe, and Asia. In Toronto the CAPS data base holds records of more than 25,000 ACHD patients. In parallel, a surgical database for all SickKids and TGH congenital heart patients was developed by 1982. As of 2024, the surgical database contained almost 23,000 patients and almost 37,000 operative records including retrospective entries for every ACHD patient since 1974. The total of ACHD surgical patients entered to date exceeds 3500. The database evolved to an on-line version in 2012 (https://toronto.ccsdb.org/) and is also used by other centres around the world.

Moreover, a highly structured standardized clinical report was developed, used by all in the ACHD clinic. This common report, revised but not recreated at each encounter, allowed easy sharing of patients, a familiar format for fellows as they moved from clinic to clinic and staff to staff, and facilitated data extraction for clinical research. It also had the virtue, demanded by the structured format, of preserving the patient's remote history and course.

## 4 Specialized clinics and programs

### 4.1 Team sport

CHD is a chronic, multisystem disorder and the management of ACHD patients calls for a team providing collaborative care (Fig. 2). How can such a team be built? In the early years, adult-trained and pediatric-trained cardiologists and congenital cardiac surgeons with interest and willingness to collaborate in the care of ACHD patients were identified. Congenital cardiac anesthesia followed, as did subspecialties of cardiology and others such as electrophysiology, intervention, pregnancy and heart disease, pulmonary hypertension, mental health, palliative care, hepatology, ACHD heart failure, genetics, and so on. As numbers of patients and complexity increased over time, some of the congenital heart subspecialties have developed dedicated clinics of their own that operate in parallel to the general ACHD clinics. The development of expertise in such areas came from interest and hands-on experience. Early on, Dr. Webb recognized the importance of a multidisciplinary approach and reached out to staff members who embraced the challenge. Dr. Louise Harris, one of the pioneering ACHD electrophysiologists globally, made significant contributions despite her initial hesitation due to a lack of specific expertise. Similarly, Dr. Jack Colman, a general cardiologist whose mid-career ACHD training consisted of once-weekly sessions for three months, did not claim expertise but showed keen interest when asked to take on the field of pregnancy and heart disease—a domain in which Toronto ultimately made substantial advancements. Selected highlights from some of these specialized programs follow.

### 4.2 Cardiac anesthesia

The perioperative care of ACHD patients is embedded in the TGH cardiovascular (CV) surgical program. In 1996 a core group of CV anesthesiologists and cardiovascular intensive care (CVICU) clinicians were assigned to ACHD peri-operative care, thus enabling a curated longitudinal experience for the patient's surgical encounter. Participation in ACHD case conferences, preoperative risk assessment and planning the intraoperative and postoperative intensive care unit management became the norm within this small subset of the CV anaesthesiology group [21]. A robust perioperative data set has been maintained since 2004 resulting in publication of quality improvement initiatives and in improved outcomes. Dr. Jane Heggie has led the group's development of a streamlined care plan for adults with neurodevelopmental and neurocognitive deficits and disorders requiring diagnostic and interventional procedures. The volume of anaesthetic needs for cardiac and non-cardiac procedures for the adult living with CHD has grown and this group act as a local and provincial resource for their perioperative care. Knowledge translation is accomplished through publications and presentations at international conferences [[22], [23], [24]]. The congenital cardiovascular anesthesia program has become a key component of and attraction for the international anesthesia fellowship program at TGH.

### 4.3 Electrophysiology

In the early years, clinical outcomes and management of ACHD patients and their arrhythmias represented “uncharted territory”. Much of the literature and management guidelines were derived from paediatric series or extrapolated from similar arrhythmias in the adult population without congenital heart disease. Together with other members of the ACHD team, the electrophysiology (EP) group undertook a major commitment to advancing and disseminating knowledge of the electrophysiologic aspects of complex congenital heart disease in the adult and conducting research to better understand the arrhythmias of this complex population.

**Fig. 4:**
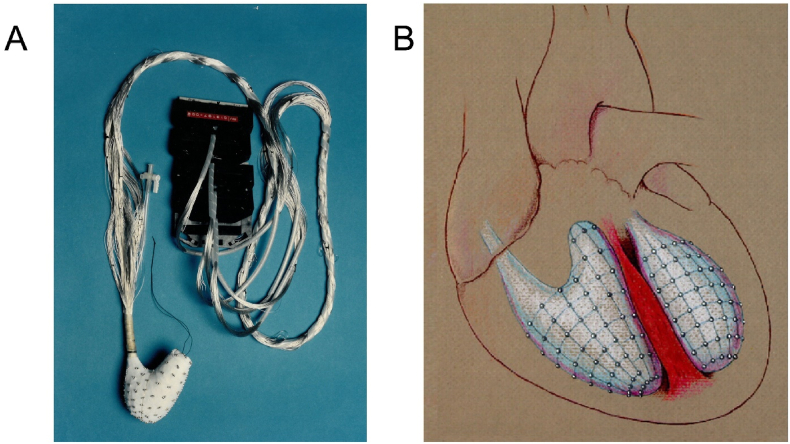
**Right Ventricular electrode array:** The right ventricular balloon electrode array was designed to enable endocardial recordings to be obtained from the body and the outflow tract of the right ventricle. One hundred and twelve electrodes were arranged in 14 rows. Following initiation of ventricular tachycardia, the re-entry circuit could be determined using this system during intraoperative mapping. This enabled the congenital heart surgeon to perform precisely targeted cryoablation of the culprit circuit. **A**. RV balloon ex vivo**. B.** Sketch of RV and LV endocardial balloons in situ for mapping.

Some of the highlights resulting from this commitment included an early intra-operative multipolar mapping system designed by Dr. Eugene Downar (cardiac electrophysiologist) in collaboration with Dr. Williams that enabled real-time mapping of ventricular tachycardia in tetralogy of Fallot, allowing surgical ablation in the era before catheter-based mapping systems and implantable defibrillators (Fig. 4) [25].

Focussing on tetralogy of Fallot, the EP team in Toronto reported for the first time that moderate to severe left ventricular dysfunction was a predictor of sudden cardiac death and established the negative impact of atrial arrhythmias on long term outcome [26,27], findings that were subsequently extrapolated to, and confirmed in, other forms of complex and moderately complex CHD [28]. Pacemakers and implantable defibrillator implants can pose unique challenges for ACHD patients as a consequence both of leads implanted in infancy, and challenging venous access or lack thereof, requiring innovative strategies. The EP team also reported at an early stage the successful application of cardiac resynchronization in a patient with previous Mustard repair and a failing systemic right ventricle [29]. Members of the EP team in Toronto have contributed to the production of various ACHD guidelines [30] and to the first guideline dedicated to arrhythmia management of ACHD patients, the PACES/HRS Expert Consensus Statement on the Recognition and Management of Arrhythmias in Adult Congenital Heart Disease [31]. From the outset, the EP group in the Toronto ACHD centre has served as a resource for arrhythmia management for those caring for these complex patients across Canada and beyond.

### 4.4 Mental health

Understanding of the prevalence in the ACHD population of mental health and neurodevelopmental and neurocognitive deficits and disorders came late to the field, occupied as CHD researchers and providers were with determining surgical and interventional solutions for complex CHD, and then discerning the unnatural medical history of the survivors. The Toronto program had the good fortune of collaboration with some of the early researchers and clinicians in ACHD mental health, including Drs. Graham Reid and Dr. Jane Irvine [32]. Dr. Adrienne Kovacs came for a clinical psychology fellowship with an ACHD focus in 2004, and then remained as staff in the Division of Cardiology and faculty in the University of Toronto Department of Psychiatry from 2006 to 2015; unique at that time, she was fully integrated and housed within the ACHD team. She was a pioneer in psychology related to acquired and especially to congenital cardiac disease, responsible for much of the early literature describing mental health challenges of this population including end-of-life issues [11,33,34]. The program thus achieved a head start in developing a formal ACHD Mental Health Program, notwithstanding the continuing challenges of ensuring adequate resources.

### 4.5 The Dalglish Family 22q clinic

The happy coincidence of a committed scientist and a motivated donor led to the establishment in 2012 of the Dalglish Family 22q Clinic, a multidisciplinary clinic founded and led by Dr. Anne Bassett of the Department of Psychiatry, who is cross-appointed in the ACHD program. This program focusses on research into and care for the multifaceted multidisciplinary manifestations of 22q11 deletion syndrome in adults. ACHD cardiologists participate in the program, offering care to patients afflicted with CHD and working alongside the multidisciplinary team who address other issues such as mental health and/or metabolic disturbances [35,36].

### 4.6 Palliative care

By 2009, maturing of the program and the patients began to make clear the need for an ACHD-aware Palliative Care focus. The field of palliative care was moving beyond its origins in end-stage cancer management at the same time, and a role was seen for palliative care principles applied earlier in the course of disease and in benign disease. “Beyond Saving Lives” was the theme of the 2012 International ACHD Symposium held in Toronto, with sessions discussing end of life questions and the provision of supportive/palliative care offered for the first time in a large ACHD forum [33,37,38]. A novel and unexpected topic at that time, surprisingly well-received nonetheless, advance care planning and application of principles of palliative care as appropriate have since become an integral part of the Toronto and many other ACHD programs [39].

### 4.7 Cardio-obstetrics

As women with repaired complex CHD began to reach adulthood in the 1980s, understanding of proper management and outcomes of pregnancy in this new and evolving population was very limited. Also, obstetricians were often fearful of managing such women through pregnancy, and a common reaction of cardiologists called to consult on pregnant women with heart disease was avoidance. Default advice was too often that pregnancy was high risk, to be avoided, and if already in progress, to be aborted. It fell primarily to ACHD cardiologists, such as Dr. Jack Colman and Dr. Samuel Siu at the outset of the Toronto pregnancy and heart disease program, to take up the challenge of determining the actual risks, defining counselling and management strategies, and dealing with complications when they occurred. Committed obstetricians such as Dr. Mathew Sermer, obstetrical anaesthetists, nurses with a special interest, all joined to constitute an effective multi-disciplinary program, a model of care now followed worldwide. The large CHD population under care in the Toronto program, and the early establishment of a pan-Canadian network of ACHD practitioners and researchers (CACHnet) made it possible for the Toronto program to lead in this area, with many papers describing natural history of individual conditions in pregnancy, and ultimately the first prospective multi-centre study of pregnancy and heart disease, the Canadian Cardiac Disease in Pregnancy (CARPREG) study [40]. CARPREG yielded a risk scoring system allowing identification prior to or in early pregnancy of women at high risk, and perhaps even more helpfully, demonstrated that those not at high risk were a larger majority than was expected a priori. Since the original CARPREG study, the Toronto program has continued to advance the understanding of pregnancy care in the ACHD population including updating the original risk assessment score (CARPREG II) [41]. Pan-Canadian collaboration in research and management of women with heart disease has resulted most recently (2021) in publication of a Clinical Practice update on Cardiovascular Management of the Pregnant Patients, sponsored by the 10.13039/100013500Canadian Cardiovascular Society and co-authored by a group all of whom were trainees and/or staff within the Toronto program at various times [42]. Cardio-obstetrics is the contemporary name for this field, still linked closely to ACHD but now a subspecialty of cardiology in its own right with expansion to include pregnant patients with acquired and arrhythmic cardiac lesions.

### 4.8 Heart failure

As the number of ACHD patients increased, it became apparent that heart failure was a leading cause of death in this population. Unfortunately, cardiologists specialized in general adult heart failure, adult cardiac transplant, and/or ACHD often feel uneasy managing such patients. Toronto researchers, including Dr Peter Liu, were some of the early investigators studying heart failure in the ACHD population [43]. In 2012, Dr. Lucy Roche established the combined ACHD-Heart Failure clinic in Toronto. The idea behind this clinic was to address the specific needs of ACHD patients with heart failure [44] and to provide a coordinated and specialized care approach [45]. The clinic accepts referrals from the ACHD team, from the heart failure and transplant teams, and from general and ACHD cardiologists across Canada. It has become the primary access point for all advanced heart failure therapies for ACHD patients in Toronto. In 2018, Dr. Rafael Alonso-Gonzalez, who had led the ACHD-heart failure program at the Royal Brompton Hospital in London, UK, joined the Toronto ACHD program and the ACHD heart failure clinic.

### 4.9 Nursing and advanced care practitioners

In nursing, as in the subspecialties of medicine, practitioners emerged who eagerly embraced the complexities and unknowns of caring for the new ACHD population. These patients often had needs and pathophysiology that differed significantly from what the nurses had previously encountered. Nurses and nurse practitioners, including pioneers like Jeanine Harrison, Marion E. McRae and Barbara Bailey, among others, joined the team. Others played crucial roles in caring for ACHD patients in the ICU or the OR. Over time, ACHD nursing evolved into a subspecialty in its own right. Nurses from the Toronto ACHD team took on teaching roles, led the formation of international ACHD nursing groups, and became integral partners for patients, families, and the entire ACHD team.

### 4.10 Research

The research enterprise of the ACHD program, first directed by Dr. Samuel Siu, later by Dr. Candice Silversides and then by Dr. Rachel Wald, has been inextricably entwined with the clinical and training program. Leveraging the large population base of the Toronto program, which received ACHD graduates from SickKids who had been early recipients of innovative surgical and interventional repairs and the adequately granular database established from the early days, much of the early research focused on observational studies that helped define the adult post-operative phenotype for lesions such as atrial septal defect [46], tetralogy of Fallot [[47], [48], [49]], Mustard procedure [50], arterial switch procedure [51], congenitally corrected transposition of the great arteries [52] and Fontan procedure [53]. Our program was among the first to report differences in behaviour and complications from the described pediatric phenotype and provided early recognition of the preponderance of complications in adults as contrasted with children and adolescents, whose course was often benign under pediatric care following recovery from the initial repairs.

## 5 Education and knowledge translation

Teams need to meet and the Toronto team, locally and nationally, meets a lot. Since 2001, ***in-patient rounds*** have taken place weekly, reviewing all admitted and recently discharged patients, thus keeping staff and fellows up to date, facilitating application of the expertise of the entire staff, and serving as a sign over. A ***weekly multidisciplinary case conference*** has been held since about 1990, at which every patient referred for surgery, complex intervention, or advanced heart failure therapies including transplant or ventricular assist device, is discussed in advance, as well as patients with obscure medical management issues. These conferences went online in 2006 as soon as the technology allowed, and rapidly expanded such that colleagues from multiple centres in Ontario and across Canada join to offer expertise and to present their own cases for multi-disciplinary guidance or as a path for referral to the Toronto congenital cardiac interventional and surgical programs. Such conferences serve as one mechanism for linking multiple centres in a “hub and spoke” arrangement such that patients can receive specialized ACHD care locally where expertise exists and still access the additional resources available in the larger centre. In addition, weekly academic rounds have been held since 2006, at first with local attendance only, and later expanded.

In the early years, management decisions were by consensus of experts. There were few data, no ACHD textbooks, no dedicated journals, and rare published articles. ***The Canadian Consensus Conference on Adult Congenital Heart Disease,*** 1996, was the first ACHD management guideline in the world [14]. It was sponsored by the Canadian Cardiovascular Society during Dr. Peter McLaughlin's tenure as president of the Society and the writing committee was chaired in Toronto by Dr. Gary Webb. Dr. Webb gathered a Canadian and international cadre of experts who reached consensus through an iterative process by e-mail and fax. The writing group never met in person. The use of email and fax in that way for that purpose in the early ‘nineties’ was pioneering. Those first Canadian guidelines served as a foundation for the first American and European guidelines [54,55]. Improvement in ACHD mortality was demonstrated following publication of the early guidelines and the increased referral to specialized ACHD centres that occurred as a result [56].

Another important goal of the Toronto program has been to bring the international AHCD community together. An ***ACHD Symposium*** was first organized in Toronto by Dr. Peter Liu in 1991, and in San Diego, California by Dr. David Sahn in 1990. By the time Dr. Sahn moved to Portland, Oregon, the magnificent and memorable Dr. Jane Somerville of the Royal Brompton in London, UK (also a Paul Wood protégée), had been guest faculty at both the Toronto and the San Diego/Portland meetings. She convinced the organizers that strength would be achieved by joining forces. That was the beginning of the annual Combined International ACHD Symposium first held at Skamania Lodge near Portland, Oregon in 1996 and then in Toronto, Ontario in 1997. The Symposium, which now alternates annually among three centres, will celebrate its 34th annual meeting in Toronto in 2025. In addition, since 2017 monthly, web-based ***Toronto International ACHD Academic Rounds*** are held and draw speakers and participants from around the world.

***Organizations and societies:*** Recognizing the need for seamless transfer of care for ACHD patients as they move from place to place, and for collaboration among ACHD practitioners across Canada for multi-centre research, Dr. Gary Webb catalyzed the formation *of* ***CACHnet*** (the Canadian Adult Congenital Heart network) in 1991. ***ISACHD*** (International Society for Adult Congenital Heart Disease) was founded in 1994, with Dr. Webb its first president. The Canadian Congenital Heart Alliance (***CCHA***), a Canadian patient and family support and advocacy organization, was founded within the Toronto patient group in 2004. In 2021, the Canadian specialty governing body, the Royal College of Physicians and Surgeons of Canada (RCPSC), approved an *Area of Focused Competence (AFC) Diploma in ACHD*, confirming ACHD as a recognized subspecialty of cardiology in Canada, the culmination of years of work by a committee chaired by Dr. Erwin Oechslin of Toronto and Dr. Luc Beauchesne of Ottawa.

## 6 The present and the future

Since its early beginning the Toronto ACHD program has grown. The program currently has 7 full-time and 2 part-time ACHD cardiologists (some with adult cardiology and some with pediatric cardiology background), an ACHD nurse practitioner, and 4 congenital cardiac surgeons who care for >12,000 active patients. In addition to the EP, Pregnancy, and Heart Failure clinics discussed above, there are several other unique clinics embedded in the Toronto ACHD program. A nurse-led Transition Program facilitated by registered nurses based both at TGH and at SickKids helps to ensure seamless transition of about 250 patients from SickKids per year, part of the 500–600 new referrals received by the program annually. About 100 surgical procedures are completed for ACHD patients per year as well as hundreds of ACHD interventions in the catheterization laboratory. A well-established Mental Health Program in ACHD is now supported by a full-time nurse practitioner and a part time psychiatrist; using our electronic medical record, we screen all our patients for mental health issues ahead of their outpatient appointments. There is a Lifestyle Program led by a sports medicine physician with the support of a part-time kinesiologist that provides exercise guidance and training to the ACHD population. Our Palliative and Supportive Care Program is enhanced by the presence of a dedicated physician for ACHD patients.

Looking to the future, our aim is to continue advancing clinical treatment strategies and launching innovative research initiatives to further improve patient outcomes and quality of life. With a focus on easing disease burden and enhancing holistic care, the program is poised to remain at the forefront of ACHD care, leveraging cutting-edge technology and interdisciplinary collaboration to address the evolving needs of this growing patient population.

## 7 The Toronto Inukshuk club

**Fig. 5:**
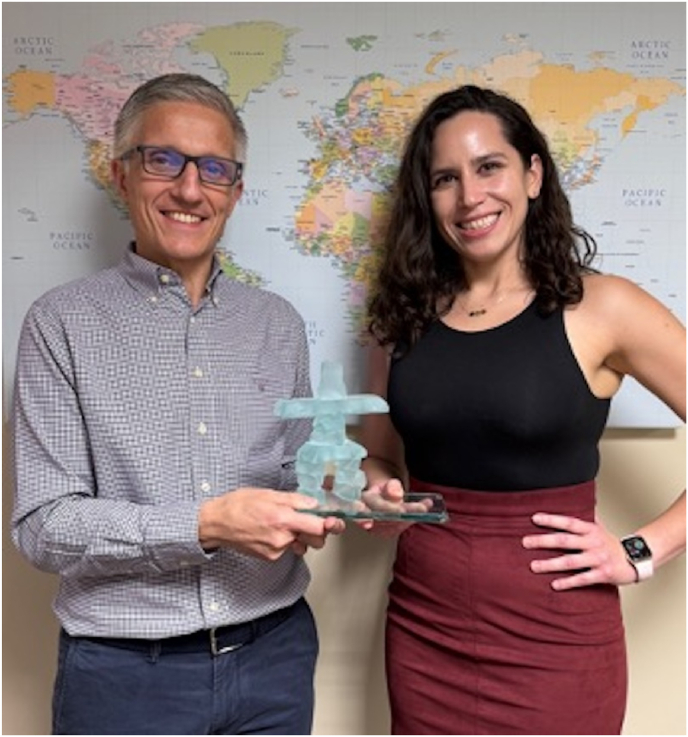
Inukshuk being presented to graduating fellow Dr. Beatriz Aldara Fernandez Campos by Dr. Rafael Alonso-Gonzalez, December 2024.

Inukshuks are stone landmarks created by the Inuit people, found in the Arctic region from Alaska through Northern Canada to Greenland. Each stone of an inukshuk is a separate entity, chosen to fit together with other stones, balanced on each other, each supporting the ones above it and supported in turn by the ones below. They serve as markers for travelers and hunters, as a warning of danger, or as a memorial. In wider Canadian culture they have become a symbol of leadership, cooperation, teamwork, interdependence, friendship and human spirit. The stones of an inukshuk achieve strength through unity. The Toronto ACHD program adopted this symbol to remind us that greater success can be achieved through cooperation and team effort than working individually. Generations of graduating ACHD fellows and departing ACHD staff have received an Inukshuk to carry its spirit on their future ACHD journey (Fig. 5).

## Dedication

We dedicate this history of the Toronto ACHD Program to the many heroic pioneer patients and families whose bravery facing the unknown and confidence in their carers, also facing many unknowns, has led. over the past 65 years, to progressive success in diagnosis and management of congenital heart disease, one of the greatest triumphs of modern medicine. More is known than at the beginning. Much remains to be learned.

## CRediT authorship contribution statement

**J.M. Colman:** Conceptualization, Data curation, Formal analysis, Project administration, Writing – original draft, Writing – review & editing. **W.G. Williams:** Data curation, Formal analysis, Writing – review & editing. **C.K. Silversides:** Data curation, Formal analysis, Writing – review & editing. **L. Harris:** Data curation, Validation, Writing – review & editing. **L. Benson:** Data curation, Validation, Writing – review & editing. **J. Heggie:** Data curation, Validation, Writing – review & editing. **R. Alonso-Gonzalez:** Conceptualization, Data curation, Formal analysis, Supervision, Validation, Writing – review & editing. **E. Oechslin:** Conceptualization, Data curation, Formal analysis, Supervision, Validation, Writing – review & editing.

## Program supports

We also acknowledge support, past and present, from the Bitove Family Foundation for the Bitove Professorship in ACHD, the Dalglish Family Foundation for support of the Dalglish Family 22q Clinic, J.W. Nevil Thomas for the Nevil Thomas Library and Nevil Thomas Fellowship, Gordon B. Allan for the Gordon B. Allan Fellowship, the Medjuck family for the Beth Medjuck Fellowship, Gregory A. Nihon for the Gregory A. Nihon Fellowship, the St. George's Society of Toronto for funding the ACHD Fellowship program, Bay Street Hockey for annual support, support for the Transition Program by an anonymous donor, and ongoing backing by the UHN Foundation.

## Funding

No funding was provided from any source in support of the development of this paper.

## Declaration of competing interest

The authors declare that they have no known competing financial interests or personal relationships that could have appeared to influence the work reported in this paper.
