# Supplementary material for: Toronto ACHD program: A 65 year legacy
Source: Int J Cardiol Congenit Heart Dis. 2024 Dec 30;19:100563. doi: 10.1016/j.ijcchd.2024.100563 (PMC11783385; doi:10.1016/j.ijcchd.2024.100563)
Supplement: Multimedia component 1 [file mmc1.docx]

**Toronto ACHD fellows 1993-2024 by country of origin**

**Europe** **37**

- United Kingdom 12
- Switzerland 8
- Spain 5
- Germany 3
- Belgium 2
- Austria 1
- Denmark 1
- France 1
- Greece 1
- Hungary 1
- Norway 1
- Sweden 1

**North America 34**

- Canada 24
- USA 8
- Mexico 2

**Central America 1**

- Honduras 1

**South America 7**

- Argentina 2
- Chile 2
- Brazil 1
- Colombia 1
- Uruguay 1

**Australia** **9**

**Caribbean 1**

- Jamaica 1

**Asia 6**

- Singapore 3
- Malaysia 1
- Philippines 1
- Thailand 1

**South Asia 7**

- India 3
- Pakistan 3
- Bangladesh 1

**Japan 3**

**Egypt** **2**

**Middle East 19**

- Saudi Arabia 9
- Israel 5
- Iran 5

**South Africa**  **1**
